# Nasal Cytology as a Local Biomarker of Airway Inflammation: A Paradigm Shift in Precision Medicine

**DOI:** 10.3390/pathophysiology33020034

**Published:** 2026-05-27

**Authors:** Matteo Gelardi

**Affiliations:** Unit of Otolaryngology, Department of Clinical and Experimental Medicine, University of Foggia, 71122 Foggia, Italy; matteo.gelardi@unifg.it

**Keywords:** nasal cytology, eosinophil–mast cell axis, type 2 inflammation, CRSwNP, clinical–cytological grading, precision medicine

## Abstract

Biomarker-driven approaches have markedly improved the stratification and management of airway inflammatory diseases. However, in everyday clinical practice, these strategies still rely mainly on systemic indicators, which often provide only an indirect view of the inflammatory processes occurring within the airway mucosa. This limitation becomes particularly evident in chronic conditions such as chronic rhinosinusitis with nasal polyps (CRSwNP), where local inflammatory patterns may not relate to circulating biomarkers. Nasal cytology represents a simple, non-invasive, and reproducible technique that allows direct evaluation of the cellular components of the nasal mucosa. By identifying distinct inflammatory patterns, it offers a real-time snapshot of the local inflammatory microenvironment, bringing the clinician closer to the site of disease. In this hypothesis, we propose that airway inflammation is primarily driven by local cytological patterns. In particular, we suggest that the interaction between eosinophils and mast cells constitutes a key pathogenic axis underlying disease activity, severity, and progression. From a pathophysiological perspective, eosinophils may reflect a more chronic component of inflammation, whereas mast cells are more closely associated with active and dynamic phases of the disease. Their coexistence may therefore identify a state of amplified inflammatory activity, often associated with more severe clinical phenotypes. We further propose that integrating cytological findings into clinical–cytological grading (CCG) systems could improve patient stratification and support more personalized therapeutic strategies. This model is readily testable in current clinical and research settings and may contribute to a progressive shift toward the use of local biomarkers in precision medicine.

## 1. Introduction

The management of airway inflammatory diseases relies on biomarker-driven approaches, which have substantially improved patient stratification. Most of these strategies are based on systemic indicators, including blood eosinophil counts, serum IgE levels, and circulating inflammatory mediators, which have played an important role in defining endotypes and guiding targeted therapies [1,2]. Nevertheless, these markers often provide only a partial and indirect picture of the inflammatory processes that occur within the airway mucosa [3].

This limitation becomes particularly evident in chronic airway diseases such as allergic and non-allergic rhinitis and chronic rhinosinusitis with nasal polyps (CRSwNP), where local inflammatory patterns may significantly diverge from systemic profiles [3,4]. At the same time, according to the concept of “united airway disease”, upper and lower airways represent interconnected compartments sharing common epithelial, immunological, and inflammatory mechanisms. Consequently, inflammatory alterations observed in the nasal mucosa may partially reflect broader airway inflammatory processes occurring throughout the respiratory tract in daily clinical practice. This mismatch may translate into suboptimal disease characterization, reduced predictive accuracy, and, ultimately, less precise therapeutic decisions [5].

For these reasons, there is increasing interest in identifying biomarkers that directly reflect the local inflammatory microenvironment [6]. Ideally, such biomarkers should be easily accessible, reproducible, non-invasive, and able to capture the dynamic nature of mucosal inflammation over time [7]. Within this scenario, nasal cytology meets many of these requirements and has progressively emerged as a valuable tool for the direct assessment of cellular components in the nasal mucosa [8,9].

By enabling the identification of different inflammatory cell populations, including eosinophils, neutrophils, mast cells, and mixed cytological patterns, nasal cytology offers what can be considered a real-time “cellular fingerprint” of airway inflammation [10]. Beyond simple cell counting, this approach allows the recognition of complex cellular interactions that are often not detectable through systemic measurements alone.

Despite its long-standing use and a well-established methodological background, nasal cytology has been relatively underutilized in routine clinical practice. This has been partly due to limited standardization in the past and to the perception of insufficient reproducibility. More recently, however, advances in methodological standardization and the integration of cytological findings into clinical frameworks have significantly improved both the reliability and the clinical relevance of this technique [11].

**Figure 1:**
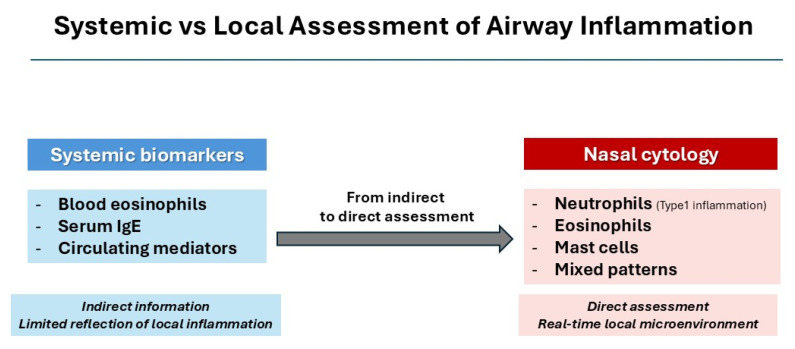
Comparison between systemic biomarkers and local cytological assessment in airway inflammation. Systemic indicators reflect circulating parameters and therefore provide only an indirect estimate of the inflammatory process. In contrast, nasal cytology enables direct visualization of the local inflammatory microenvironment.

In this perspective, we propose that nasal cytology should be viewed not only as a diagnostic tool, but as a dynamic local biomarker capable of complementing conventional approaches and providing a direct, clinically meaningful assessment of the airway inflammatory microenvironment (Figure 1).

This shift in perspective may lead to a more accurate phenotyping of patients and better support the development of precision medicine approaches in rhinology and beyond.

## 2. Current Understanding of Airway Inflammation

Airway inflammatory diseases are characterized by a complex and heterogeneous network of immune responses involving both innate and adaptive immunity. Within this spectrum, type 2 inflammation has emerged as a dominant paradigm in a substantial proportion of patients with allergic and non-allergic airway diseases, including CRSwNP and asthma. This inflammatory pattern is mainly driven by T helper 2 (Th2) lymphocytes and group 2 innate lymphoid cells (ILC2), which orchestrate the production of key cytokines such as interleukin (IL)-4, IL-5, and IL-13 [5].

Eosinophils have long been regarded as the hallmark effector cells of type 2 inflammation [12]. Their recruitment, activation, and persistence within the airway mucosa are largely mediated by IL-5 and eotaxins, contributing to tissue damage, mucus hypersecretion, and remodeling processes [3]. As a result, eosinophil counts in peripheral blood and tissue have been widely used as surrogate biomarkers of disease severity and as criteria for therapeutic decision-making, particularly in the context of biologic therapies targeting the IL-5 pathway [1].

However, accumulating evidence indicates that eosinophil-centered models do not fully capture the complexity of airway inflammation [13]. In clinical practice, a number of patients show a clear discordance between blood eosinophil levels and the degree of local tissue inflammation [14,15]. Moreover, considerable clinical heterogeneity persists even among patients with comparable eosinophilic profiles [5,13], suggesting that additional cellular and molecular mechanisms are involved.

For these reasons, increasing attention has been directed toward other immune cell populations, including mast cells, neutrophils, and epithelial cells, which actively contribute to the orchestration and amplification of inflammatory responses [16]. Among these, mast cells deserve particular consideration. Beyond their well-known role in early-phase allergic reactions, they are also involved in chronic inflammation through the release of preformed mediators such as histamine and proteases, as well as cytokines and growth factors, and through their interaction with other immune cells [16].

The interaction among different cellular components within the airway mucosa creates a dynamic and spatially organized inflammatory microenvironment that cannot be fully captured by systemic measurements alone [17,18]. This complexity highlights the need for approaches capable of directly assessing local cellular interactions and identifying distinct inflammatory endotypes beyond simplified classifications [13].

Overall, these observations suggest that current models of airway inflammation, although highly informative, remain incomplete and could be strengthened by integrating tools that allow direct evaluation of the local inflammatory population.

## 3. Nasal Cytology: A Direct Window into Local Inflammation

Nasal cytology is a well-established, non-invasive, and easily repeatable technique that allows direct evaluation of the cellular components of the nasal mucosa. Performed through a simple nasal scraping, it enables the collection of epithelial and inflammatory cells, which can then be analyzed using standardized staining protocols, most commonly those of May–Grünwald–Giemsa. The sampling procedure is typically performed under anterior rhinoscopy by gently scraping the middle portion of the inferior turbinate using a sterile disposable curette or nasal scraper. This area is considered particularly suitable because it provides a representative balance between epithelial and inflammatory cells while minimizing patient discomfort. The procedure is painless, rapid, and easily repeatable during follow up. This method, derived from hematological cytology, provides excellent visualization of cellular morphology and allows reliable identification of different inflammatory cell populations [6].

A key strength of nasal cytology lies in its ability to offer a direct and real-time assessment of the local inflammatory microenvironment. Unlike systemic biomarkers, which reflect circulating mediators or peripheral cell counts, nasal cytology captures the actual cellular composition at the site of inflammation. This distinction is particularly relevant in airway diseases, where local processes frequently do not mirror systemic findings. Importantly, nasal cytology should not be interpreted as a direct surrogate of lower airway pathology. Rather, it represents a minimally invasive window into airway inflammation, capable of providing indirect but clinically meaningful information regarding the inflammatory status of the respiratory tract, particularly in type 2 inflammatory diseases.

Through cytological analysis, distinct inflammatory patterns can be recognized, including eosinophilic, neutrophilic, mast cell–dominant, and mixed cellular profiles. These patterns reflect different pathogenic mechanisms and may correspond to specific clinical phenotypes. In this sense, nasal cytology is not merely descriptive, but represents a practical tool for defining inflammatory endotypes directly at the mucosal level.

The interpretation of airway inflammation has traditionally been shaped by histopathological approaches based on hematoxylin–eosin staining. While this technique has been essential for identifying tissue eosinophilia, it has also contributed to an eosinophil-centered view of airway inflammation. Eosinophils are readily detectable because of their characteristic staining properties, whereas mast cells are often underestimated or even overlooked due to their limited visibility with routine histological staining [9].

In contrast, May–Grünwald–Giemsa staining, routinely used in nasal cytology, allows clear identification of mast cells through their metachromatic granules, making it possible to detect inflammatory patterns that would otherwise remain unrecognized. This difference is not only technical, but also carries important pathophysiological implications.

From a functional perspective, eosinophils are typically associated with chronic and persistent inflammation, reflecting a relatively stable phase of the disease. Mast cells, on the other hand, through their rapid degranulation and release of potent mediators, are more closely linked to active and dynamic phases of inflammation. Their coexistence within the same cytological sample may therefore indicate a state of amplified inflammatory activity, potentially associated with increased disease severity and progression [19].

These considerations suggest that the predominance of eosinophils in traditional models of airway inflammation may, at least in part, reflect a methodological bias rather than a complete representation of the underlying biological processes. By revealing cellular components that are less evident with conventional histology, nasal cytology provides a more comprehensive and nuanced view of the inflammatory landscape.

### Limitations of Nasal Cytology

Despite its advantages, nasal cytology also presents some limitations. The quality of the results depends on an adequate sampling technique, proper staining procedures, and operator expertise in cytological interpretation. In addition, although methodological standardization has significantly improved in recent years, variability among centers may still exist. Furthermore, nasal cytology primarily reflects superficial and local mucosal inflammation and does not directly assess deeper tissue remodeling. Nevertheless, its simplicity, repeatability, low cost, and ability to provide real-time information continue to make it an attractive tool in both clinical practice and research settings.

Overall, these features support the concept of nasal cytology as a true “cellular fingerprint” of airway inflammation, offering a unique opportunity to directly observe the local inflammatory milieu and refine current pathophysiological models [9].

## 4. The Hypothesis

### A Local Cytological Axis as a Driver of Airway Inflammation

Current models of airway inflammation largely rely on systemic biomarkers and histopathological assessments, which have shaped an eosinophil-centered interpretation of disease mechanisms. While these approaches have provided important insights, they do not fully capture the complexity and dynamic nature of the local inflammatory microenvironment.

Building on the direct cellular assessment offered by nasal cytology, we hypothesize that airway inflammation is primarily driven by local cytological patterns, which may reflect disease activity and severity more accurately than systemic indicators alone. In particular, we propose that the interaction between eosinophils and mast cells represents a key pathogenic axis underlying disease expression, severity, and progression.

Within this framework, eosinophils can be viewed as markers of a chronic and persistent inflammatory component, whereas mast cells appear to be more closely associated with active, dynamic, and potentially exacerbating phases of the disease. The coexistence of these two cell populations within the nasal mucosa may therefore identify a state of amplified inflammatory activity, associated with greater clinical severity and an increased risk of disease progression.

This hypothesis challenges the traditional eosinophil-centered paradigm and supports a broader interpretation of airway inflammation, in which mast cells are considered active contributors rather than secondary players. From a clinical standpoint, this model can be tested directly by correlating cytological patterns with clinical outcomes, disease severity scores, and response to targeted therapies.

If confirmed, this framework may support a shift from systemic to local biomarkers in the assessment of airway diseases, improving patient stratification and enabling more precise and personalized therapeutic strategies. This perspective may be particularly relevant in CRSwNP, a condition in which disease heterogeneity continues to complicate clinical decision-making.

In this context, integrating cytological findings into a grading may provide a more immediate and accessible tool for patient stratification [20]. Such an approach could guide a stepwise therapeutic strategy, ranging from conventional medical treatments to biologic therapies selected according to the underlying inflammatory profile.

Beyond treatment selection, this stratification may also offer a rational basis for treatment modulation over time. In particular, a cytology-driven approach could help to identify patients suitable for dose reduction or interval adjustment of biologic therapies, an area that remains insufficiently explored but is increasingly relevant for long-term disease management and healthcare sustainability. Ultimately, this strategy may help optimize therapeutic efficacy while reducing unnecessary treatment burden and associated costs.

## 5. The Eosinophil–Mast Cell Axis

The inflammatory landscape of airway diseases is shaped by a complex interplay among multiple immune cell populations. Within this network, eosinophils and mast cells represent two key effectors of type 2 inflammation. Although they have traditionally been studied in parallel, their functional relationship has often been underestimated.

Eosinophils are widely recognized as central mediators of chronic airway inflammation [21,22]. Their accumulation within the mucosa is associated with tissue remodeling, epithelial damage, and mucus hypersecretion [22,23]. These features are typically linked to persistent and relatively stable phases of the disease, which has led to the widespread use of eosinophils as primary biomarkers in both clinical and research settings [21,24].

Mast cells, on the other hand, have long been viewed mainly as early-phase effector cells involved in allergic reactions. However, growing evidence suggests that their role extends well beyond the initial phases of inflammation. Mast cells can release a wide range of preformed and newly synthesized mediators, including histamine, tryptase, chymase, cytokines, and growth factors, thereby contributing not only to the initiation but also to the amplification and maintenance of inflammatory responses [19].

**Figure 2:**
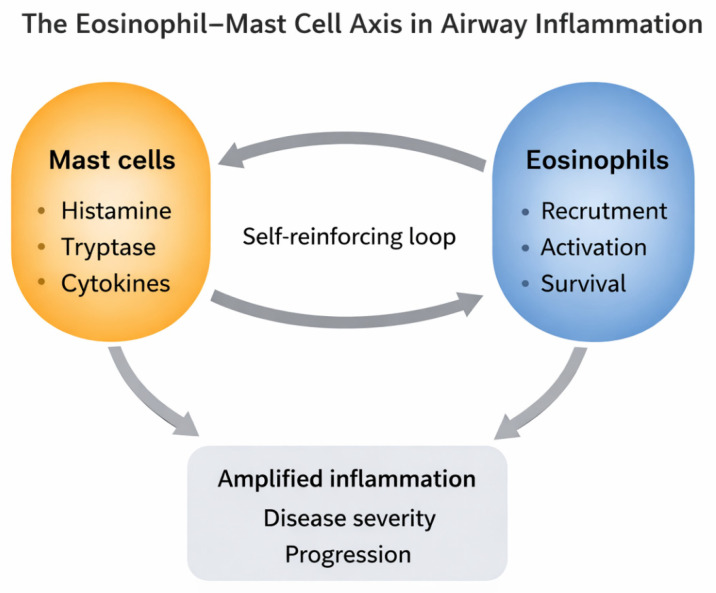
Schematic representation of the eosinophil–mast cell axis. The reciprocal interaction between mast cells and eosinophils generates a self-sustaining inflammatory loop, in which each cell population amplifies the activity of the other, ultimately contributing to disease activity, severity, and progression. Arrows indicate bidirectional cellular interaction and amplification of inflammatory signaling.

Importantly, eosinophils and mast cells do not operate in isolation. Instead, they engage in a dynamic and reciprocal interaction within the airway mucosa [25]. Mediators released by mast cells can promote eosinophil recruitment and activation, while eosinophils, in turn, can support mast cell survival and function through the release of cytokines and growth factors [26]. This bidirectional crosstalk establishes a self-reinforcing inflammatory loop, which may enhance both the intensity and persistence of the disease [27] (Figure 2).

From a cytological perspective, the coexistence of eosinophils and mast cells within the same sample defines a distinct inflammatory pattern [28], which may reflect a state of increased biological activity. This observation is particularly relevant in CRSwNP, where mixed eosinophil–mast cell infiltration has been associated with greater disease severity and a higher risk of recurrence, as previously reported [29].

Within this framework, the eosinophil–mast cell axis may represent a transition state between chronic inflammation and active disease exacerbation. Eosinophils appear to reflect the persistence of type 2 inflammation, whereas mast cells are more closely linked to ongoing and dynamic processes characterized by mediator release and tissue reactivity. Their coexistence may therefore identify patients with a more aggressive inflammatory profile [30].

Taken together, these findings suggest that airway inflammation should not be interpreted solely on the basis of eosinophil presence or absence, but rather through an integrated evaluation of multiple cellular components and their interactions. In this context, nasal cytology provides a unique opportunity to detect and characterize these interactions in vivo, offering insights that are not accessible through systemic biomarkers or conventional histopathology.

## 6. Clinical Translation: From Cytology to Precision Medicine

The integration of nasal cytology into clinical practice offers a concrete opportunity to translate cytological patterns into clinically actionable information. By directly assessing the local inflammatory milieu, nasal cytology provides a level of detail that goes beyond conventional clinical evaluation and systemic biomarkers, allowing a more accurate characterization of the disease.

The identification of distinct cytological profiles enables a more refined stratification of patients. Eosinophilic patterns are typically associated with chronic type 2 inflammation, whereas the presence of mast cells may indicate a more active and dynamic inflammatory phase. In particular, mixed eosinophil–mast cell profiles may identify patients with a more aggressive disease phenotype, often characterized by greater severity, higher recurrence rates, and variable response to treatment.

The integration of cytological findings with clinical parameters has contributed to the development of combined clinical–cytological grading systems, such as Clinical–Cytological Grading (CCG) [20], aimed at improving patient stratification and better reflecting the complexity of airway inflammation. By incorporating comorbidities, including allergy, asthma, and aspirin sensitivity, together with cytological patterns, this approach allows a more comprehensive assessment of disease burden. As such, it provides a practical framework for translating cellular information into clinically meaningful patient stratification.

From a therapeutic perspective, nasal cytology may support more personalized treatment strategies. Identifying the predominant inflammatory pattern can help guide therapeutic choices according to the underlying endotype, with the potential to optimize treatment response. In addition, given its non-invasive and repeatable nature, cytology can be used as a dynamic tool to monitor disease evolution and assess therapeutic efficacy over time [31].

More broadly, this cytology-based approach aligns with the principles of precision medicine, which aim to move beyond standardized treatment algorithms toward individualized patient management. In this context, nasal cytology may serve as a bridge between pathophysiological mechanisms and clinical decision-making, enabling a more targeted and adaptive approach to airway diseases.

Overall, the incorporation of nasal cytology into routine clinical assessment may improve diagnostic accuracy, enhance risk stratification, and support the development of more personalized therapeutic strategies in patients with airway inflammatory diseases.

## 7. Testability of the Hypothesis

### Clinical Implications of the Hypothesis

A fundamental requirement of any hypothesis-driven model is that it can be tested within current clinical and research frameworks. The concept of nasal cytology as a dynamic local biomarker, together with the proposed role of the eosinophil–mast cell axis in defining disease activity and severity, lends itself to direct evaluation through several complementary approaches.

Cross-sectional studies may explore the relationship between cytological patterns and established indicators of disease severity, including symptom scores, endoscopic findings, and radiological staging. In this setting, patients with mixed eosinophil–mast cell infiltration could be compared with those showing isolated eosinophilic or non-eosinophilic patterns, in order to identify differences in clinical presentation and overall disease burden.

Longitudinal studies offer an additional and particularly informative perspective. Repeated nasal cytology assessments could be used to follow disease evolution over time, helping to determine whether the presence of mast cells is associated with periods of increased inflammatory activity, exacerbations, or progression toward more severe phenotypes.

The hypothesis can also be tested by examining the relationship between cytological profiles and response to therapy. Patients could be stratified according to their inflammatory pattern and evaluated in relation to their response to topical treatments, systemic therapies, or biologic agents targeting type 2 inflammation. This approach may help clarify whether the eosinophil–mast cell axis identifies specific responders or non-responders.

In addition, comparative analyses between systemic biomarkers and local cytological findings may provide further insight into the degree of concordance, or discordance, between circulating indicators and mucosal inflammation. Such studies could strengthen the concept that local assessment offers more accurate and clinically relevant information.

Importantly, all these approaches can be implemented using currently available diagnostic tools and within standard clinical practice, without the need for complex or invasive procedures. The simplicity, repeatability, and low cost of nasal cytology make it particularly well suited for integration into both clinical research and routine care.

Overall, these considerations indicate that the proposed hypothesis is not only conceptually sound, but also readily testable, offering a practical framework for future studies aimed at validating the role of nasal cytology in precision medicine.

## 8. Concluding Remarks

The current paradigm of airway inflammation, largely grounded in systemic biomarkers, has led to important advances in disease understanding and management. However, this framework does not fully account for the complexity and spatial heterogeneity of the inflammatory processes occurring within the airway mucosa.

In this article, we propose a shift in perspective toward the use of nasal cytology as a dynamic local biomarker, capable of providing a direct and integrated view of the inflammatory microenvironment. By identifying distinct cytological patterns, and particularly the interaction between eosinophils and mast cells, nasal cytology offers insight into both the chronic and active phases of airway inflammation.

The concept of the eosinophil–mast cell axis as a driver of disease expression and severity supports a more nuanced interpretation of type 2 inflammation, moving beyond simplified and predominantly eosinophil-centered models. This view emphasizes the importance of cellular interactions and local dynamics as key elements of disease pathophysiology.

This model is readily testable within current clinical and research settings and may have direct implications for patient stratification, therapeutic decision-making, and disease monitoring. In this sense, the integration of cytological assessment into clinical practice may help bridge the gap between pathophysiological mechanisms and precision medicine.

An additional issue that deserves attention is the limited dissemination of nasal cytology in routine clinical practice, partly due to insufficient training during medical education. Despite its simplicity, reproducibility, and clinical relevance, cytological assessment of the nasal mucosa is still not systematically included in the training programs of key specialties involved in airway diseases, including otolaryngology, allergology, pulmonology, and pediatrics.

Incorporating nasal cytology into residency curricula and continuing medical education programs could represent a crucial step toward its broader adoption. Improving clinicians’ familiarity with cytological techniques and interpretation would facilitate the integration of local inflammatory assessment into everyday clinical practice, ultimately enhancing patient management and supporting the transition toward precision medicine.

In conclusion, prioritizing the direct evaluation of the local inflammatory microenvironment represents a meaningful step forward in the management of airway inflammatory diseases. By providing a real-time cellular fingerprint of mucosal inflammation, nasal cytology has the potential to reshape current diagnostic and therapeutic approaches, moving toward a more personalized and biologically grounded model of care.

## Data Availability

No new data were created or analyzed in this study. Data sharing is not applicable to this article.
